# Finite element analysis of occlusal splint therapy in patients with bruxism

**DOI:** 10.1186/s12903-019-0897-z

**Published:** 2019-09-04

**Authors:** Seifollah Gholampour, Hanie Gholampour, Hamed Khanmohammadi

**Affiliations:** 1 Department of Biomedical Engineering, North Tehran Branch, Islamic Azad University, Tehran, Iran; 2 Department of Electrical and Computer Engineering, Science and Research Branch, Islamic Azad University, Tehran, Iran

**Keywords:** Occlusal splint therapy, Bruxism, Stress, Deformation, Temporomandibular joint (TMJ), Finite element method (FEM)

## Abstract

**Background:**

Bruxism is among the habits considered generally as contributory factors for temporomandibular joint (TMJ) disorders and its etiology is still controversial.

**Methods:**

Three-dimensional models of maxilla and mandible and teeth of 37 patients and 36 control subjects were created using in-vivo image data. The maximum values of stress and deformation were calculated in 21 patients six months after using a splint and compared with those in the initial conditions.

**Results:**

The maximum stresses in the jaw bone and head of mandible were respectively 4.4 and 4.1 times higher in patients than in control subjects. Similar values for deformation were 5.8 and 4.9, respectively. The maximum stress in the jaw bone and head of mandible decreased six months after splint application by up to 71.0 and 72.8%, respectively. Similar values for the maximum deformation were 80.7 and 78.7%, respectively. Following the occlusal splint therapy, the approximation of maximum deformation to the relevant values in control subjects was about 2.6 times the approximation of maximum stress to the relevant values in control subjects. The maximum stress and maximum deformation occurred in all cases in the head of the mandible and the splint had the highest effectiveness in jaw bone adjacent to the molar teeth.

**Conclusions:**

Splint acts as a stress relaxer and dissipates the extra stresses generated as well as the TMJ deformation and deviations due to bruxism. The splint also makes the bilateral and simultaneous loading possible and helps with the treatment of this disorder through regulation of bruxism by creating a biomechanical equilibrium between the physiological loading and the generated stress.

## Background

In adults, the prevalence of temporomandibular disorders is 25-50% and, in particular, the prevalence of bruxism is 8-31.4% [1, 2]. In general, habits such as bruxism are contributory factors for temporomandibular joint (TMJ) disorders. From a biomechanical point of view, TMJ is the most complex joint in the human body. More than 2000 neuromuscular control signals are registered daily for normal performance of this joint [3]. The consequences of bruxism are revealed mostly in form of wear and damage and are more prevalent in men than women [4]. Bruxism not only leads to wear, grinding, crushing, fracture and ultimately serious damages to teeth but also may cause hearing loss, maxillofacial problems and even facial deformation [5]. If bruxism is not treated, the teeth, bones and gum may be worn or fractured due to wear pressure [6]. Since bruxism is the most important risk factor for TMJ [7], the study of suitable strategies for treating this disorder is of great importance.

Previous studies related to the subject of this research can be divided into two main groups. The first group of studies has examined merely the changes in the biomechanical parameters in TMJ and mandible. Tanaka et al. examined the effect of age on the manner of changes in the parameters affecting the TMJ disc displacement [8]. Hirose et al. investigated the destructive effects of prolonged jaw and teeth pressing on TMJ disc using finite element method (FEM) [9]. Donzelli et al. examined the kinematic and geometric changes in TMJ discs using FE analysis [10]. Koolstra et al. showed with the help of FEM that the articular disk has the ability to distribute loads in a wide area [11]. In a biomechanical study, Naeije et al. investigated the loads exerted on TMJ during chewing and chopping [12]. Del Palomar et al. examined with the help of FEM the effective biomechanical parameters in lateral excursions of the mandible during chewing [13]. Commisso et al. examined the effect of pterygoid muscles on movement of the jaw during mastication using FE analysis [14]. Nishigawa et al. measured the maximum bite force in patients with sleep associated bruxism experimentally, however, they did not deal with the change of bite force during or after treatment [15]. Some studies focused solely on biomechanical parameters affecting implant insertion and filling teeth and dental pain [16–19].

The second group of studies has focused on the assessment of treatments of TMJ disorders. Ferreira et al. showed how the occlusal splint distributes stress in TMJ disc [20]. Salmi et al. found a new digital process to produce occlusal splints in a study using a laser scanner and evaluated the effectiveness of this new production method [21]. Kobayashi et al. examined the association between masticatory performance and bite force in children with bruxism [22].

The results of previous studies have shown that occlusal splint therapy can be an option for treatment of patients suffering from bruxism [22–24]. These studies showed that biomechanical factors such as stress are associated with bruxism, however the exact contribution of these parameters is still unknown [22]. Therefore, it was tried in this research to perform a study with the aim of quantitative assessment of the effectiveness of occlusal splint for treating bruxism using in vivo image data of patients and control subjects. The manner of changes in biomechanical parameters affecting this disease, such as stress and deformation, after occlusal splint therapy was also examined in the present study in order to gain more insight into the detail performance mechanism of this therapy.

## Methods

A total number of 37 volunteers were selected from the 420 patients who had been referred to the North Tehran Dental Hospital during 23 months. The patients included 19 women and 18 men aged between 21-49 years old and with a body mass index between 18. 2-21.9 kg.m^− 2^. 5.5% of male and 10.5% of female patients had in up to 3 teeth a history of tooth caries and tooth fracture which did not result from jaw or mouth injury and were treated previously and did not pose a particular problem during the study. Further, 55.5% of male and 52.6% of female patients had a history of dental filling in up to 2 teeth. All patients were evaluated first using a questionnaire in order to identify the main complaint, pain history and bruxism [25, 26]. It should be noted that this research was approved by the Ethics Committee of the North Tehran Branch of the Islamic Azad University (No. 18245/86–2) in accordance with the 1964 Helsinki declaration. In this study, all participants provided informed consent according to the ethical standard. An equally standardized diagnostic protocol was applied by a professional dentist for all patients before and after the occlusal splint therapy. This diagnostic protocol included an interview and a systematic evaluation of dental, cranial, facial, cervical and other oral structures [25, 26]. No craniofacial surgery, no use of any medication and no reported systemic disease were the initial inclusion criteria. Selection of patients with sleep bruxism was done based on the criteria of American Academy of Sleep Medicine as follows [27]:

A. Occurrence of tooth grinding at least 3 nights per weeks for 6 months, as confirmed by a sleep partner; B. clinical presence of tooth wear; C. hypertrophy of masseter muscle; and D. occurrence of fatigue or tenderness of jaw muscle in the morning.

From the Subjects referred to the North Tehran Dental Hospital, 36 volunteers included 18 women and 18 men aged between 26-52 years old and with a body mass index of 19. 6-22.3 kg.m^− 2^, who had no history of disease or symptoms associated with bruxism or TMJ disorders, were selected as control subjects. They were completely healthy with regard to bruxism or TMJ disorders, but had toothache from a cracked or attritioned tooth (22.2% of males and 16.6% of females), tooth fracture and tooth caries in up to 4 teeth (27.8% males and 38.8% female). 66.7% of males and 77.8% of females from the control subjects needed a dental implant for up to 4 teeth. During the diagnosis and treatment process, CT scans were done based on the specialist’s diagnosis. Also 61.1% of males and 55.5% of females from the control subjects had a history of dental filling in up to 3 teeth. It should be noted that the control subjects underwent all stages of diagnosing process and informing about this study the same as the patients, and provided informed consent as well.

Cone-beam CT (CBCT) scanning was used for preparing images due to its low costs, unique accessibility and low effective radiation dose. A Newtom VG system (QR, Verona Italy) was used for CBTC scan setting. The scan setting included: 3.6 mAs and 90 KV with radiation time of 15 s and field of view of 20 × 19 in.. Furthermore, the subjects were in standing position during scanning and their head was in natural head position. Patients were asked not to swallow or breathe during imaging. The voxel size and slice thickness were 0.3 × 0.3 × 0.3 mm. and 0.3 mm, respectively. It should be noted that for all patients and control subjects, the jaw was in the maximum intercuspation position during imaging.

**Fig. 1:**
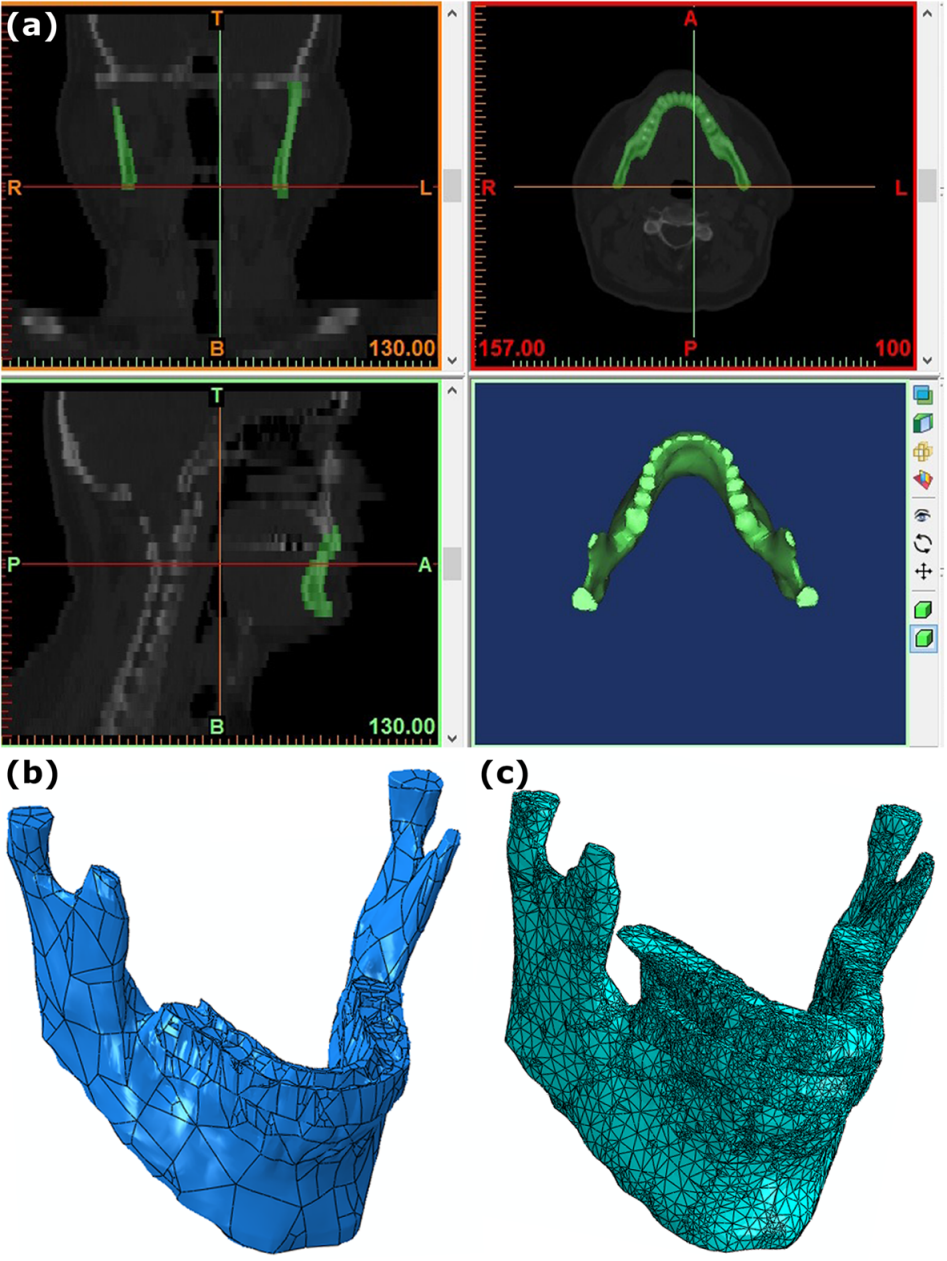
**a** The point clouds of the maxilla, mandible and teeth. **b** 3D model. **c** Meshed model

The CT scan images of the subjects’ jaw and teeth of were used and the DICOM files of these images were imported into Mimics software version 13.1 (Materialise, Leuven, Belgium) for producing the point clouds of the maxilla, mandible and teeth of each subject as separate parts (Fig. 1a). In Mimics, the bony parts of the maxilla, mandible and teeth in image file were kept. After modifying the areas containing soft tissue in all image slices and repeating these modifications layer by layer, the spaces between layers were finally modified and differentiated and the point clouds of the teeth and jaw bone were extracted as the software output. Subsequently, the point clouds were transferred to the CATIA software version 5R21 (Dassault Systemes, Waltham, Mass., USA), and a three-dimensional model of the maxilla, mandible and teeth was built (Fig. 1b).

Three-dimensional models of the maxilla, mandible and teeth of all 37 patients and 36 control subjects were assembled in conditions of no contact pressure with respect to each other. Six months after insertion of the occlusal splint, the process of CT scanning, creating the point cloud, and building the three-dimensional models of jaws, teeth and splint were repeated for patients with the splint in their mouth. At this stage, the 3D models of patients’ splints were constructed from their CT scan images and the 3D splint models were inserted between the patients’ upper and lower teeth. It should be noted that all patients were treated using occlusal splint therapy based on the diagnosis of an experienced dentist. However, sleep hygiene measures combined with relaxation techniques were advised and prescribed for all patients. The material of splints was a hard colorless acrylic resin polymerized using the conventional heat-curing method. It is worth noting that due to personal limitations of the patients, it was only possible to create the 3D model of jaw, teeth and splint of 10 women and 11 men after 6 months of using splint. Since the analyses based on FEM are among the most familiar methods for biological simulation [28–41], the assembled models were transferred finally to ABAQUS software version 6.14 (Dassault Systemes) for FE analysis. Furthermore, for comparing the results of computer simulations in patients and control subjects and also for comparing the results in patients before and after the occlusal splint therapy, three specific anatomical points of the skull of samples were used as set points to synchronize the procedures and to standardize the samples’ head position. The 3D models of the samples were standardized in terms of defining the x, y, and z axis with respect to the same reference in order to ensure the correct loading distribution for comparing the biomechanical parameters of the models with each other.

**Fig. 2:**
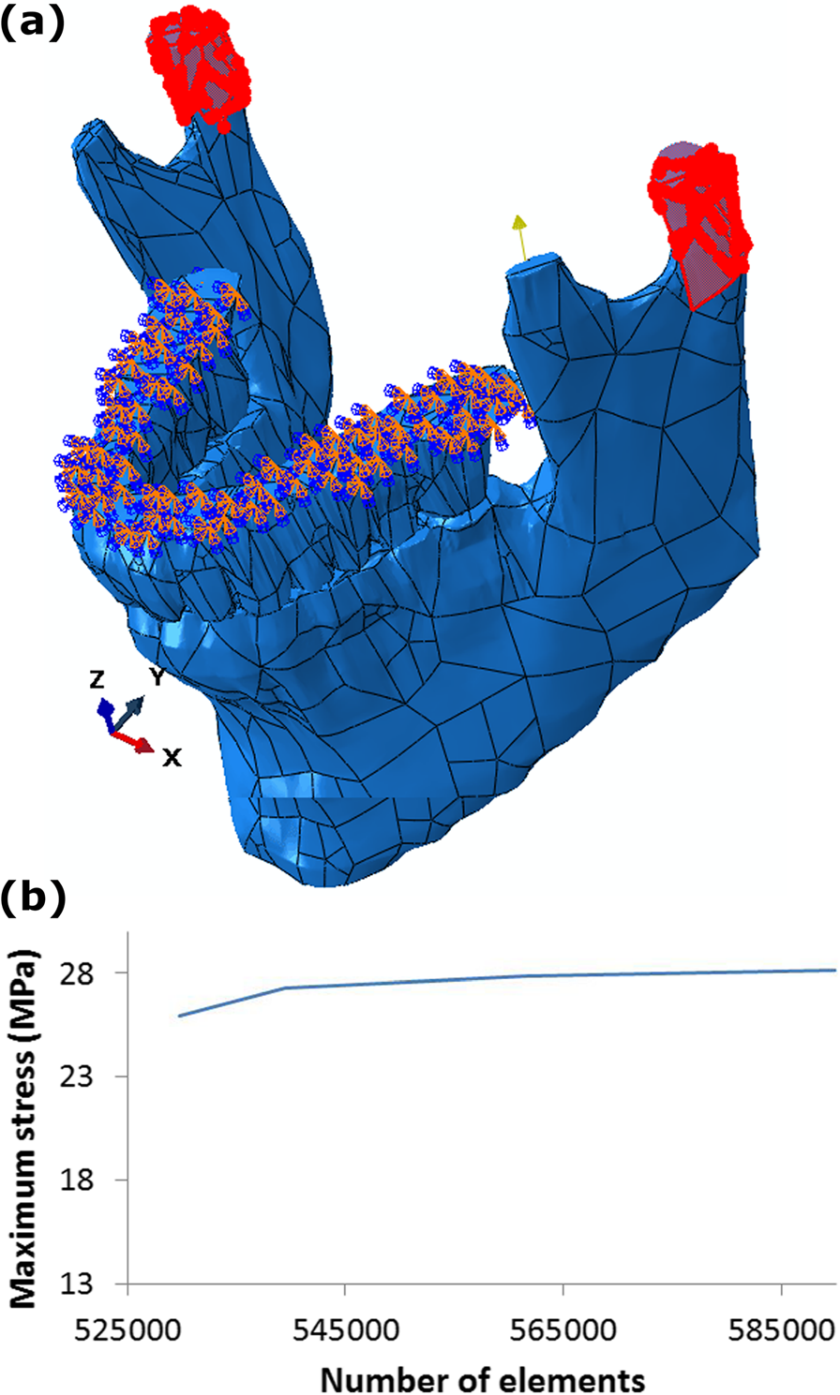
**a** Degree of freedom, interactions and constraints between the parts. **b** Diagram of the maximum stress in the head of mandible – the number of elements for grid independence study

Table 1 shows the material properties considered for jaw bones, teeth, and splints [20, 42]. One of the most important points in FE analysis is how different parts of the model interact with each other. In this study, these interactions and constraints were defined based on the actual anatomical function of these components in human body. The constraint considered for the contact between the inserted teeth on the upper and lower jaws with splint was a surface-to-surface constraint with a friction coefficient of 0.5 [20]. The degree of freedom of the upper surface of maxilla was considered to be zero at all three directions of x, y and z, i. e., the surface was considered to be fixed. Other degrees of freedom were considered in accordance with the real performance of TMJ, so that the necessary degrees of freedom for opening and closing movements of jaw (rotational degree of freedom) as well as the translation and lateral displacement of jaws over each other (translational degree of freedom) were considered (Fig. 2a). According to a previous study, a first order Ogden hyperelastic model was used for defining the periodontal ligament with a Poisson’s ratio of 0.45 and the material parameter **μ = 0.0025** MPa [43]. The average force exerted by the medial pterigoid muscle and masseter muscle for both of the left and right muscles was assumed to be 50 N [20, 42]. It should be noted that, according to previous studies, the force of these muscles should be applied under a particular angle to the model, as shown in Fig. 2a. One of the most important issues in numerical computer simulations is to ensure the mesh convergence of responses [44–48]. The tetrahedral element was used for meshing the models (Fig. 1c). The results showed that the maximum difference between the stress values in the medium and fine meshes in all three groups of patients, control subjects and patients after using the occlusal splint for 6 months was less than 1.8%. Therefore, the convergence of responses from the grid and time step was ensured (Fig. 2b).

**Table 1:** Material properties of jaw bone, teeth and occlusal splint [20, 42]

Parameters	Elastic modules (MPa)	Poison ratio
Jaw bone	1370	0.3
Teeth	18,000	0.31
Occlusal splint	0.027	0.35

### Statistical analyses

The mean value, standard deviation (SD) and coefficient of variation (CV) for maximum stress and maximum deformity were calculated using SPSS version 22 (IBM Corp., Armonk, New York, USA) in all three groups of patients, control subjects and patients after using the occlusal splint for 6 months.

## Results

**Fig. 3:**
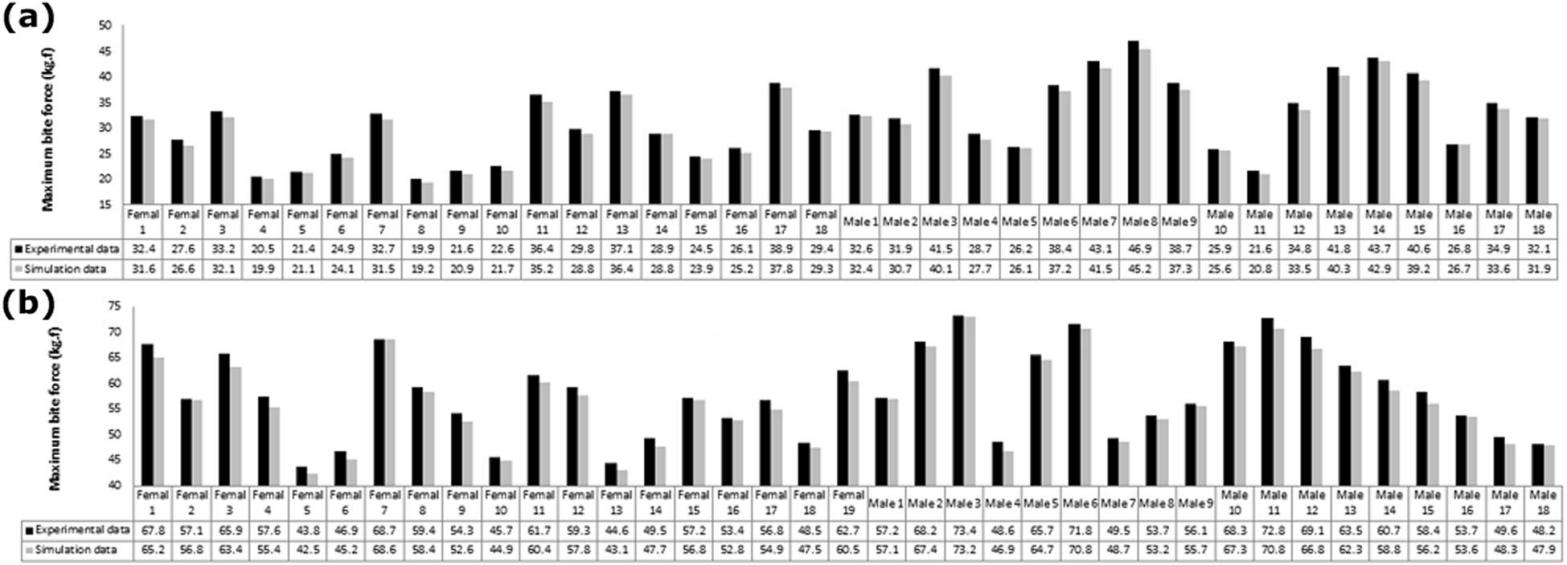
The maximum difference between the computer simulation and the experimental results of the maximum bite forces in control subjects **a** and patients **b**

Data validation is one of the most important concerns in computer simulations. Therefore, it is necessary to verify the correctness of the simulation results in order to assure the accuracy of the assumptions and modeling and analysis processes. For data validation, the maximum bite force was measured experimentally in control subjects and patients (before using the splint) and was compared with the similar results calculated in the simulation process. For this goal, a miniature strain-gauge transducer (LM-50- KAM186, Kyowa Electronic Instruments Co., Tokyo, Japan) was mounted on the first molar regions right and left in all subjects and the average value of maximum bite forces was registered after 30 records for each subject and was compared with the similar parameter calculated by FEM simulation. Figure 3 shows that the maximum difference between the computer simulation and experimental results of the maximum bite forces in all patients and control subjects was less than 3.9%. Therefore, the validity of the simulation process is confirmed. Then, the correctness of the statistical analysis of the main results of this study, i.e. the maximum stress and maximum deformation, should be assured for each of the three groups of patients, control subjects and patients after using the splint for 6 months. The results of the statistical analysis showed that the highest amount of CV in all three groups of subjects was less than 3.1% for both maximum stress and maximum deformation (Table 2). The results in Table 2 showed that SD and CV values ​​were acceptable for all parameters in all three groups of subjects. It should be noted that the reported values ​​for maximum stress and maximum deformation in the rest of the paper are the average values of these parameters for each of the three groups of patients, control subjects and patients after using the splint for 6 months.

**Table 2:** Stress and deformation values in jaw bone and head of mandible. SD: standard deviation; CV: coefficient of variation

Cases	Values	Jaw bone			Head of mandible		
		Maximum	SD	CV	Maximum	SD	CV
Control subjects	Stress (MPa)	4.82	0.14	3.01	6.91	0.18	2.82
	Deformation (× 10^-4^ mm)	63.91	1.61	2.70	9.4	0.23	2.68
Patients	Stress (MPa)	21.10	0.63	3.10	28.26	0.81	3.07
	Deformation (× 10^-4^ mm)	368.10	10.31	3.06	437.2	13.64	3.10
Patients 6 months after using splint	Stress (MPa)	6.12	0.16	2.82	7.68	0.18	2.56
	Deformation (× 10^-4^ mm)	71.10	1.97	2.91	93.11	2.65	3.05

**Fig. 4:**
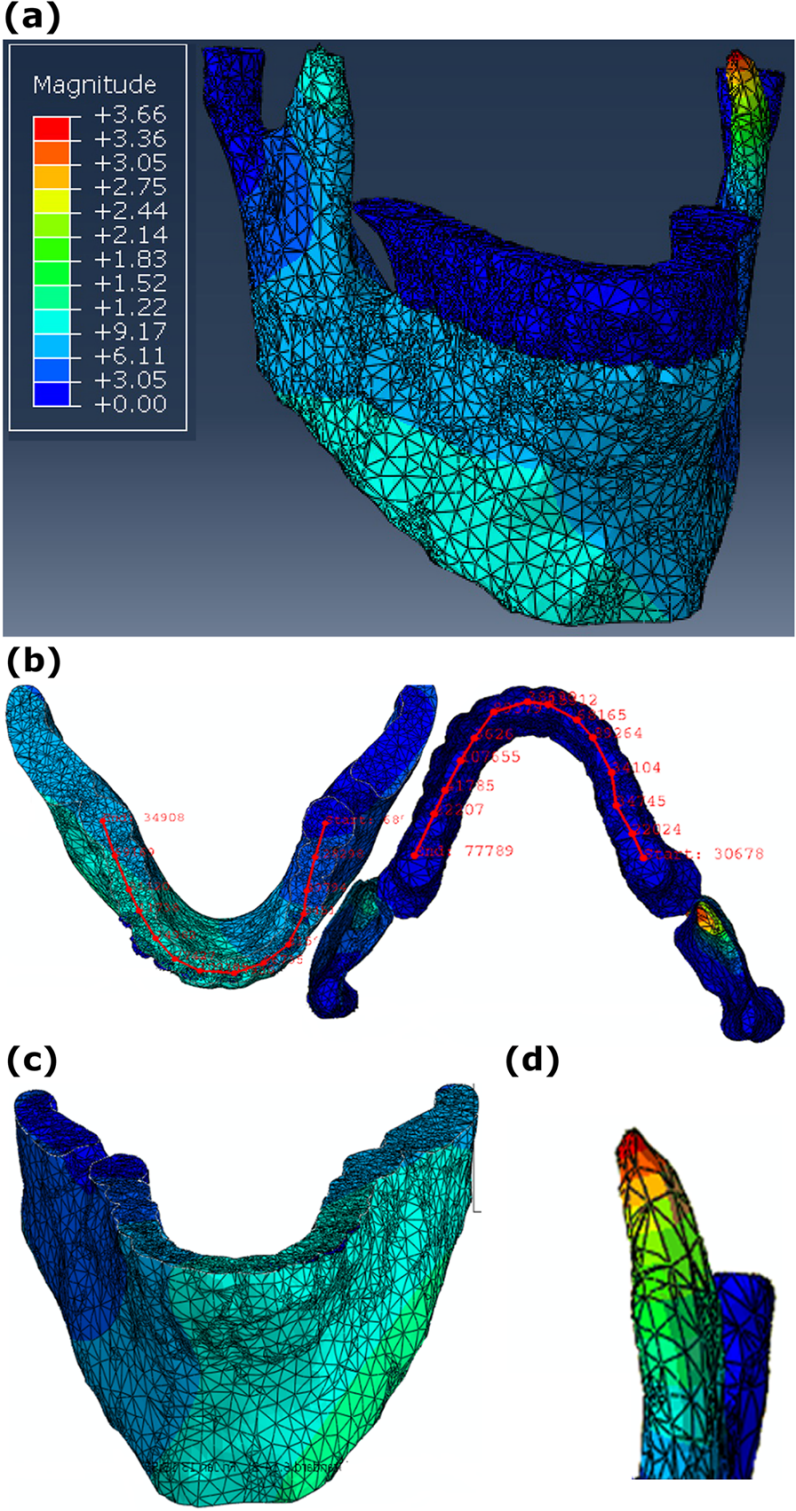
**a** Stress distribution of the jaw bone in a control subject **b** the manner of changes in deformation values in the upper and lower jaws. **c** Deformation distribution of the jaw bone in a control subject. **d** The location of maximum stress

According to Fig. 4 a and Table 2, the maximum stress in the jaw bone of control subjects and patients was 4.82 and 21.10 MPa, respectively. Results of analysis of patients 6 months after using the splint showed that the maximum stress was 6.12 MPa. The maximum stress produced in the head of mandible of control subjects and patients was 6.91 and 28.26 MPa, respectively. The respective value of this parameter in patients 6 months after using the splint was 7.68 MPa.

Figure 4, b shows the manner of changes in deformation values. The results showed that the maximum deformation in the jaw bone of control subjects and patients was 63.91× 10^-4^ and 368.10× 10^-4^ mm (Fig. 4 c and Table 2). Similarly, the result for patients six months after using the splint was 71.10× 10^-4^ mm. The maximum deformation in the head of mandible of control subjects and patients was 9.40× 10^-4^ mm and 437.20× 10^-4^ mm, respectively. This value decreased in patients 6 months after using the splint to 93.11× 10^-4^ mm (Table 2).

## Discussion

The main objectives of this study were the numerical examination of the manner of changes in effective parameters in occlusal splint therapy during bruxism treatment as well as the assessment of the effectiveness of using occlusal splint for treating this disease. Stress and deformation are the most important biomechanical indices for assessing TMJ disorders and are usually used for quantitative examination of these disorders [14, 49]. However, the bite force, as mentioned earlier, was used for data validation in the present study due to the fact that the experimental measurement of stress and deformation is very difficult [14] and the experimental measurement of the distribution of these parameters in the jaw bone is impossible. Therefore, the bite force parameter, which can be obtained both experimentally and using FEM simulation, was used in this study for data validation. The values of the von Mises stress and deformation in the jaw bone were also calculated using FE method in patients, control subjects and patients 6 months after using the splint in order to assess the disease. The results showed that the maximum stress in the jaw bone of patients was 4.4 times the maximum stress ​​in control subjects. After using the splint, the maximum stress decreased by 71.0% (Table 2). However, based on the results, the maximum stress in patients after using the splint did not return exactly to the range of maximum stress in control subjects and there was a difference of 26.9% between the values of maximum stress ​​in control subjects and patients after splint treatment. The contact surfaces opposite to the jaw bone are not exactly parallel to it and there are various geometric complexities in this area. Also, with regard to the jaw anatomy, the muscular forces exerted to it are not exactly perpendicular to the surface and are exerted in a particular orientation. Therefore, the von Mises stress calculated by computer simulation presented shear stress, in addition to compressive stress. Consequently, all areas undergoing deformation are not in the same direction and this can also lead to local shear stress. Hence, the lateral walls of the splint can play an effective role in confrontation with these shear stresses and this is an important issue in designing the splint. Regarding the large difference between the elastic modules of the splint material and the jaw bone and the teeth materials (Table 1), a large contribution to load absorbing and damping can be attributed to the splint due to the softness and flexibility of its material. Therefore, the splint acts as an absorber and dissipater of the generated stress and can help reduce and somehow relax the stress. If this additional loading due to the bruxism is not damped by the splint, a reaction force and consequently an additional reaction stress will be generated in TMJ, which will damage the joints, muscles and ligaments associated with TMJ. Therefore, splint creates a biomechanical equilibrium between the physiological loading and the generated stress through stress relaxation. The imbalance between the input physiological loading and the generated stress can be one of the biomechanical causes of bruxism and the splint can contribute to the neuromuscular reflex and to reduce the stresses on the ligaments and joints associated with the TMJ by helping achieve this balance. As shown in Fig. 4 d, the maximum stress in the total jaw bone is generated in the TMJ and, in particular, in the head of mandible. Its biomechanical cause can be the stress concentration, since the head of mandible, considering the jaw bone anatomy, has the most complex and limited cross sections in the entire mandible. The maximum stress in the head of the mandible in patients was 4.1 times that in control subjects but reduced by 72.8% after using the splint. The difference between this stress and the similar parameter in control subjects was 11.1%. The important point is that 6 months after using the splint, the difference between the maximum stress in the head of the mandible and the other parts of the jaw bone was closer to the similar values in control subjects. This means that the effectiveness of the occlusal splint in patients suffering from bruxism in reducing the stress in the head of mandible is higher than other locations of mandible. It should be noted that the location of generation of the maximum stress after using the splint did not change and the maximum stress always occurred in the head of mandible. The results showed that the highest deformation, like the maximum stress, occurred in the head of the mandible. According to Table 2, the maximum deformation in the jaw bone and head of mandible of patients was, respectively, 5.8 and 4.9 times that in control subjects and decreased by 80.7 and 78.7% six months after using splint. The difference between the maximum deformation in the jaw bone and head of mandible of patients who used splint for six months and control subjects was 11.3 and 4.1%, respectively. The results in Table 3 show that the maximum stress and deformation in all subjects were greater in the lower jaw than in the upper jaw. Similar to the reported results for patient No. 1, the greatest effectiveness of the splint in the jaw bone was related to the areas adjacent to the first, second and third molar teeth in all patients (Table 3). The results also showed that the stresses in the left and right mandible of patients are not necessarily balanced and uniform. However, due to the flexibility of the splint material, the bilateral and simultaneous loading becomes possible, which can also be useful in the treatment of bruxism.

**Table 3:** Stress and deformation values in upper and lower teeth of patient No. 1 and patient No. 1 after using splint

Tooth number		Maximum stress in upper teeth (kPa)		Maximum stress in lower teeth (kPa)		Maximum deformation in upper teeth (× 10^-5^ mm)		Maximum deformation in lower teeth (× 10^-5^ mm)	
		Patient	Patient after using splint	Patient	Patient after using splint	Patient	Patient after using splint	Patient	Patient after using splint
Left side	Third molar	0.56	0.17	1121.3	63.1	0.023	0.004	25.4	5.6
	Second molar	0.34	0.09	923.6	58.2	0.023	0.004	23.8	5.4
	First molar	0.26	0.09	854.7	55.6	0.021	0.003	21.9	5.4
	Second premolar	0.11	0.08	487.6	54.3	0.016	0.002	14.7	4.8
	First premolar	0.08	0.07	364.8	54.1	0.011	0.002	13.6	4.8
	Canine	0.21	0.18	784.6	754.5	0.016	0.014	21.8	21.4
	Lateral incisor	0.18	0.08	728.4	54.5	0.016	0.003	21.8	5.1
	Central incisor	0.17	0.07	526.8	54.3	0.015	0.003	15.9	5.1
Right side	Third molar	0.62	0.19	1264.3	68.9	0.028	0.004	24.9	5.5
	Second molar	0.61	0.17	1026.7	62.8	0.025	0.003	24.1	5.5
	First molar	0.49	0.11	830.2	59.4	0.025	0.003	20.8	5.2
	Second premolar	0.28	0.08	377.6	53.4	0.016	0.002	15.2	4.9
	First premolar	0.08	0.06	362.9	52.1	0.015	0.001	12.5	4.7
	Canine	0.36	0.32	810.6	794.2	0.025	0.024	19.7	19.5
	Lateral incisor	0.23	0.08	710.8	58.8	0.023	0.003	19.4	5.1
	Central incisor	0.19	0.08	649.7	55.4	0.022	0.001	15.5	5.0

It should be noted that the minimal effectiveness of the splint in damping the stress and deformation was related to the canine teeth. The results showed that the occlusal splint therapy was effective in reducing stress and deformation, especially in the head of mandible. It should be noted that following the occlusal splint therapy, the maximum deformation approached almost 2.6 times the maximum stress to the respective value in control subjects. Thus, the effectiveness of splint was higher in reducing deformation than stress. In fact, the design of the occlusal splint therapy is not based on the prevention of bruxism, but the results of this study show that the occlusal splint can help treat this disease by reducing stress and correcting deformations and deviations, especially in the head of mandible, and eventually reducing the additional support reaction due to bruxism in TMJ. It is suggested that the future studies record and compare the stimulation of TMJ-related muscles affecting bruxism before and after using splint by patients, so that the effect of splint on muscle stimulation and bruxism frequency is also examined as the main focus of the present study was on intensity not frequency.

## Conclusion

The results showed that the occlusal splint creates a biomechanical equilibrium between the physiological loading and the generated stress through stress relaxation. The splint also provides the possibility for making the asymmetric and non-uniform loading due to bruxism bilateral and simultaneous. Thus, the occlusal splint can lead to regulation of bruxism by reducing stresses, and in particular, by reducing deformations and deviations in TMJ and consequently can help treat this disease. The results of this study can be useful in quantitative evaluation of the changes in stress and deformation before and after treatment of bruxism as well as in development of a biomechanical approach for assessing the effectiveness of occlusal splint therapy.

## Data Availability

All relevant data are within the present paper; however, the ethics committee strictly forbids sharing the CBCT files of patients and control subjects that they contain some identifying information, according to its regulations on the data access. Therefore, CBCT data of the samples will not be shared.

## Abbreviations

CV: coefficient of variation
FEM: finite element method
SD: standard deviation;
TMJ: temporomandibular joint
